# 18α-Glycyrrhetinic Acid Induces Apoptosis of HL-60 Human Leukemia Cells through Caspases- and Mitochondria-Dependent Signaling Pathways

**DOI:** 10.3390/molecules21070872

**Published:** 2016-07-01

**Authors:** Yi-Chang Huang, Chao-Lin Kuo, Kung-Wen Lu, Jen-Jyh Lin, Jiun-Long Yang, Rick Sai-Chuen Wu, Ping-Ping Wu, Jing-Gung Chung

**Affiliations:** 1 Department of Biological Science and Technology, China Medical University, Taichung 404, Taiwan; YCH@mail.cmu.edu.tw; 2 Department of Chinese Pharmaceutical Sciences and Chinese Medicine Resources, China Medical University, Taichung 404, Taiwan; clkuo@mail.cmu.edu.tw (C.-L.K.); JLY@mail.cmu.edu.tw (J.-L.Y.); 3 College of Chinese Medicine, School of Post-Baccalaureate Chinese Medicine, China Medical University, Taichung 404, Taiwan; prorna@mail.cmu.edu.tw; 4 Division of Cardiology, China Medical University Hospital, Taichung 404, Taiwan; pig222@ms15.hinet.net; 5 Department of Anesthesiology, China Medical University Hospital, Taichung 404, Taiwan; rickwu@mail.cmuh.org.tw; 6 Department of Medicine, China Medical University, Taichung 404, Taiwan; 7 School of Pharmacy, China Medical University, Taichung 404, Taiwan; 8 Department of Biotechnology, Asia University, Taichung 413, Taiwan

**Keywords:** 18α-glycyrrhetinic acid, mitochondria, caspase-3, apoptosis, HL-60 cells

## Abstract

In this study we investigate the molecular mechanisms of caspases and mitochondria in the extrinsic and intrinsic signal apoptosis pathways in human leukemia HL-60 cells after in vitro exposure to 18α-glycyrrhetinic acid (18α-GA). Cells were exposed to 18α-GA at various concentrations for various time periods and were harvested for flow cytometry total viable cell and apoptotic cell death measurements. Cells treated with 18α-GA significantly inhibited cell proliferation and induced cell apoptosis in a dose-dependent manner, with an IC_50_ value of 100 μM at 48 h. The cell growth inhibition resulted in induction of apoptosis and decreased the mitochondria membrane potential (ΔΨ_m_) and increased caspase-8, -9 and -3 activities. Furthermore, cytochrome c and AIF were released from mitochondria, as shown by western blotting and confirmed by confocal laser microscopy. Western blotting showed that 18α-GA increased the levels of pro-apoptotic proteins such as Bax and Bid and decreased the anti-apoptotic proteins such as Bcl-2 and Bcl-xl, furthermore, results also showed that 18α-GA increased Fas and Fas-L which are associated with surface death receptor in HL-60 cells. Based on those observations, the present study supports the hypothesis that 18α-GA-induced apoptosis in HL-60 cells involves the activation of the both extrinsic and intrinsic apoptotic pathways.

## 1. Introduction

Leukemia is a heterogeneous group of hematopoietic malignancies [1] and the sixth leading cause of cancer-associated death, causing 7.1 deaths per 100,000 persons per year in the USA [2]. In Taiwan, where 4.2 per 100,000 persons die annually from the disease based on the 2012 report from the Department of Health, Executive Yuan, Taiwan [3] leukemia is the 13th leading cause of cancer-associated death. Acute leukemia are clonal disorders of hematopoietic stem cells that can be divided into acute myeloid leukemia (AML) which can occur at all ages and acute lymphoid leukemia (ALL) which most often occurs in children and adolescents, with approximately 60% of patients being younger than 20 years [4,5,6]. It was reported that about 50% of the young adult patients and approximately 90% of older patients with AML or ALL die from those diseases [5,6]. Currently, the cure-rate for patients with AML remains at approximately 25% [7], mainly due to the side effects which still are the major limit to treatment success. Thus, finding new compounds derived from natural products for leukemia is needed.

Induction of cancer cell apoptosis has been recognized to be one of the best strategies for anticancer agent activity. Apoptosis, a programmed cell death, can be divided into extrinsically and intrinsically mediated pathways. Both pathways involve the activation of caspases (cysteine proteases) for inducing cell apoptosis [8,9]. The extrinsically-activated pathway involves the activation of cell surface ligand-gate death receptors such as Fas and Fas-L and triggering of the pro-caspase-8 to form active-caspase-8 followed by activation of caspase-3 to induce apoptosis [10,11]. The intrinsically-activated pathway, also called the mitochondria-mediated pathway, through mitochondrial dysfunction, leads to cytochrome c release into the cytoplasm and induction of apoptosome formation with Apaf-1 and the activation of caspases for inducing apoptosis [12,13,14]. Furthermore, pro-apoptotic protein Bax, the ratio of Bak and anti-apoptotic protein Bcl-2 and Bcl-xl also play an important role affecting the mitochondrial dysfunction involved in the mitochondria-dependent apoptosis pathway [15,16].

Currently, about 47.1% of approved anticancer drugs are unmodified natural products, their semisynthetic derivatives, or molecules synthesized based on natural products [17]. Glycyrrhetic acid (GA), a component of the plant *Glycyrrhiza uralensis*, exhibits in vivo biological activities such as hepatoprotective, anti-inflammatory and anticancer functions [18,19,20]. The mechanisms of GA’s anticancer activities have been known to involve reduced cell proliferation, induced cell cycle arrest and apoptosis and the inhibition of cellular metastasis [19,21,22]. 18α-Glycyrrhetinic acid (18α-GA), a hydrolyzed metabolite of glycyrrhizin, had been shown to display anti-inflammatory, antiviral and anticancer activities [18,23,24,25]. It was reported that 18α-GA inhibits the proliferation of activated hepatic stellate cells (HSCs) and induces apoptosis and increases peroxisome proliferator-activated receptor-γ (PPAR-γ) expression and decreases NF-κB DNA-binding activity [26]. Glycyrrhetinic acid (GE) (a hydrolysis product of glycyrrhizic acid) had been shown to prevent or to induce the mitochondrial permeability transition (a phenomenon related to oxidative stress) in rat heart mitochondria depending on its concentration [27]. Recently, it was reported that 18α-GA inhibited gingival fibroblast growth by suppressing the G1/S phase transition and inducing apoptosis [28]. However, there is no available information to show the effects of 18α-GA on human leukemia cells, thus, the aim of present study was to investigate the in vitro toxic effects of 18α-GA on human leukemia HL-60 cells. The results showed that 18α-GA treatment of HL-60 cells resulted in apoptotic cell death through mediation by extrinsically and intrinsically mediated pathways.

## 2. Results

### 2.1. 18α-GA-Induced Cytotoxic Effects on Human Leukemia HL-60 Cells

**Figure 1:**
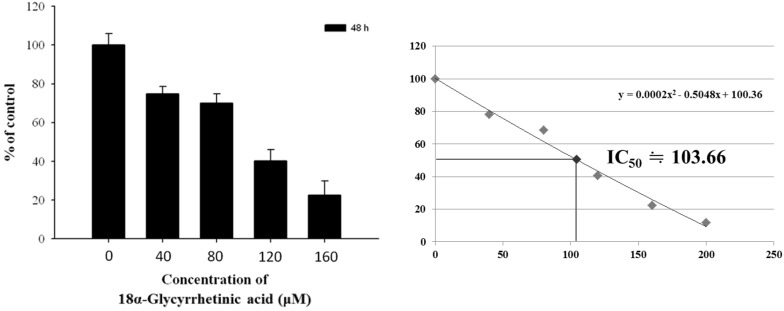
18α-GA induced cytotoxic effects on human leukemia HL-60 cells. HL-60 cells (1 × 10^4^ cells/well) were placed onto 96-well plated in 100 μM of RPMI-1640 medium. The medium was containing 18α-GA (0, 40, 80, 120 and 160 μM) for 48 h. Percent of cell proliferation was measured by MTT assay as described in the Materials and Methods section.

The cytotoxic effects of 18α-GA on HL-60 cells were evaluated by MTT colorimetric assay with the results shown in Figure 1. 18α-GA displayed the strongest HL-60 cell growth inhibition potency, accompanied by a reduction of viable cells and this inhibition is dose-dependent. The calculated IC_50_ for 18α-GA on HL-60 cells was 100 μM. Thus, we selected this concentration for further experiments.

### 2.2. 18α-GA Induced Nuclear Condensation, DNA Damage and Fragmentation in HL-60 Cells

**Figure 2:**
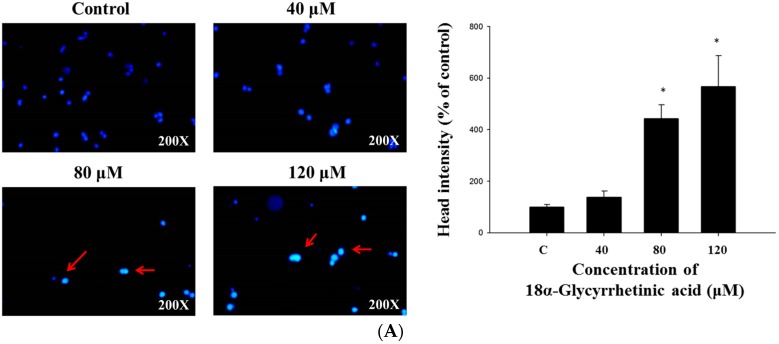

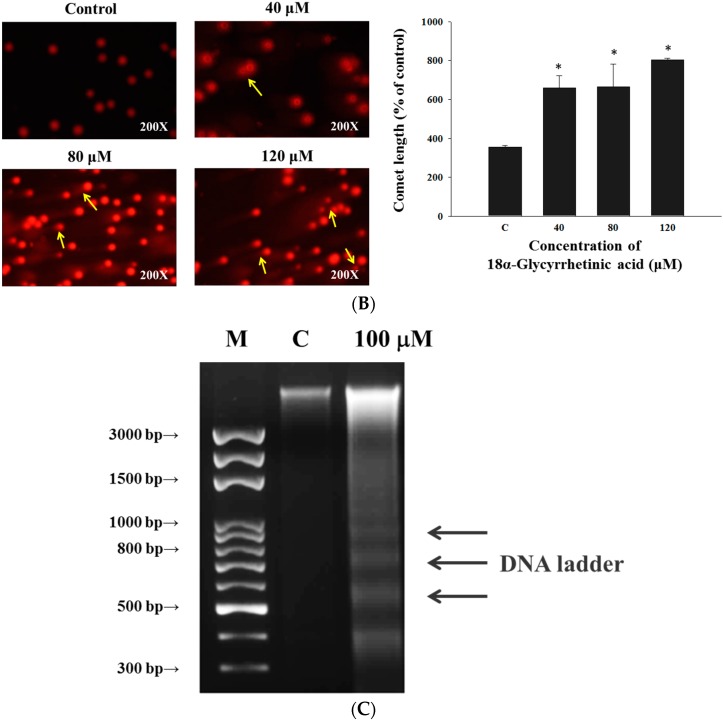
18α-GA induced nuclear condensation, DNA damage and fragmentation in HL-60 cells. HL-60 cells were treated with 18α-GA (0, 40, 80 and 120 μM) for 48 h. Cells were stained with DAPI for measuring nuclear condensation (**A**) or cells were performed by Comet assay for DNA damage evaluated (**B**); Cells were treated with 100 μM of 18α-GA for 48 h and DNA was isolated for gel electrophoresis for examining DNA fragmentation (**C**) as described in the Materials and Methods section. * *p* < 0.05, significant difference between 18α-GA-treated groups and control as analyzed by Student’s *t* test.

HL-60 cells were treated with 18α-GA (0, 40, 80 and 120 μM) for 48 h. Cells were stained with DAPI for measuring nuclear condensation (Figure 2A), or cells were subjected to a Comet assay for DNA damage evaluation (Figure 2B). The results indicated a brighter fluorescence of HL-60 cells after 48 h treatment of 18α-GA at 40–120 μM (Figure 2A) and this bright fluorescence represents nicked DNA and nuclear chromatin condensation. The results also indicated a comet tail for HL-60 cells after 48 h treatment of 18α-GA at 40–120 μM (Figure 2B) and the longer comet tail represents DNA damage. Both effects are dose-dependent manner. Cells were treated with 100 μM of 18α-GA for 48 h and DNA was isolated for gel electrophoresis with results shown in Figure 2C, where 18α-GA at 100 μM clearly induced a typical ladder pattern of internucleosomal fragmentation (DNA fragmentation). DNA fragmentation is a hallmark of cell apoptosis, thus this finding suggests that 18α-GA induces apoptosis of HL-60 cells.

### 2.3. 18α-GA Decreased the Levels of Mitochondrial Membrane Potential (ΔΨm) and Increased the Activities of Caspase-8, -9 and -3 in HL-60 Cells

**Figure 3:**
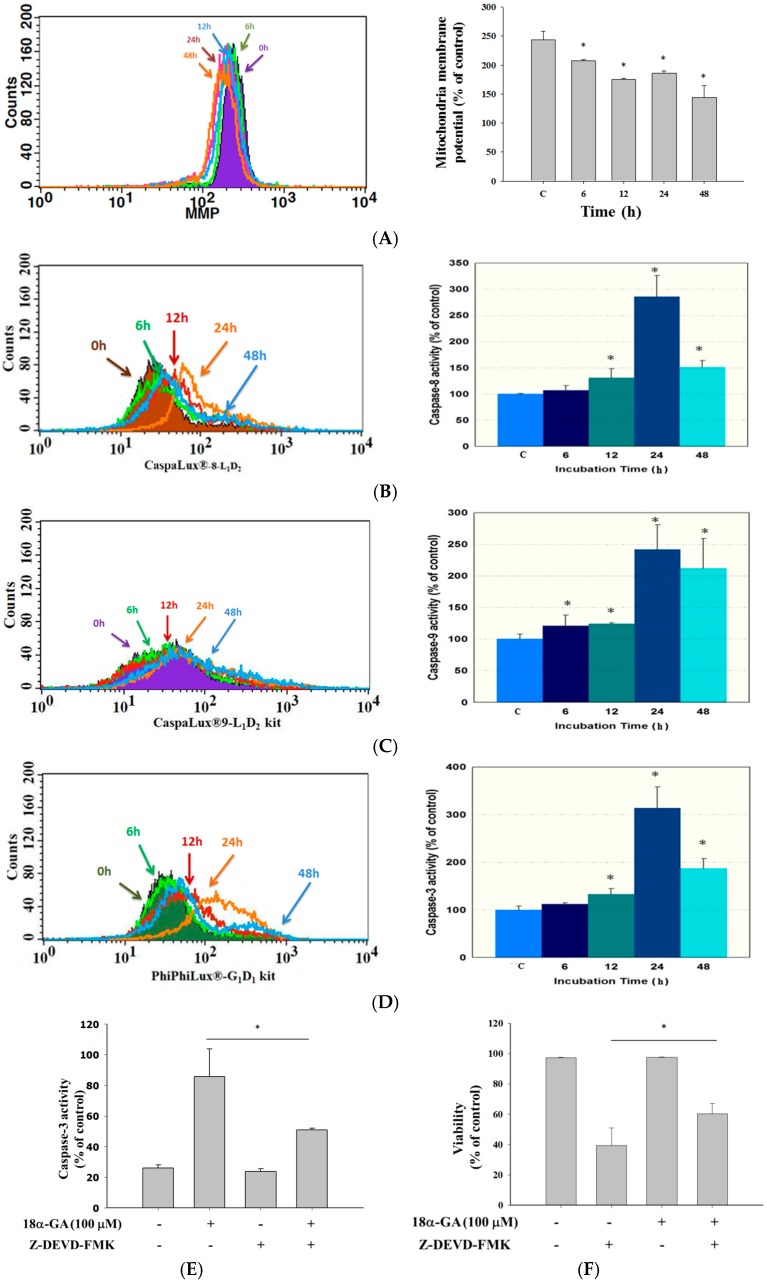
18α-GA decreased the levels of mitochondrial membrane potential (ΔΨm) and increased the activities of caspase-8, -9 and -3 in HL-60 cells. Cells were treated with 18α-GA (100 μM) for 6, 12, 24 and 48 h and were analyzed by flow cytometric assay for the levels of mitochondrial membrane potential (ΔΨm) (**A**); caspase-8 (**B**); caspase-9 (**C**) and caspase-3 (**D**) activities. Cells were pretreated with z-DEVD-FMK (caspase-3 inhibitor) and then treated with 18α-GA for measuring the activities of caspase-3 (**E**) and cell viability (**F**). * *p* < 0.05, significant difference between 18α-GA-treated groups and the control as analyzed by Student’s *t* test.

In order to confirm that 18α-GA induced cell apoptosis through the dysfunction of mitochondria and activations of caspase-8, -9 and -3 in HL-60 cells, cells were treated with 18α-GA (100 μM) for 6, 12, 24 and 48 h and were analyzed by flow cytometric assay and the results are shown in Figure 3. 18α-GA decreased the levels of mitochondrial membrane potential (ΔΨm) from 6 h to 48 h treatment (Figure 3A) when compared to untreated groups. These results indicated that ΔΨm are involved in 18α-GA induced cell apoptosis in HL-60 cells in vitro. The results showed that 18α-GA increased caspase-8 (Figure 3B), -9 (Figure 3C) and -3 (Figure 3D) activities compared to untreated groups, thus, caspase-8, -9 and -3 are involved in 18α-GA induced cell apoptosis of HL-60 cells in vitro. Cells were pretreated with caspase-3 inhibitor (z-DEVD-FMK) for 1 h and then treated with 18α-GA and the results shown in Figure 3E,F indicated that 18α-GA increased the total viability but decreased the activities of caspase-3 when compared to untreated groups. Based on these findings, it indicated that 18α-GA induced cell apoptosis through the activation of caspase-3.

### 2.4. 18α-GA Altered Apoptosis Associated Protein Expression in HL-60 Cells

**Figure 4:**
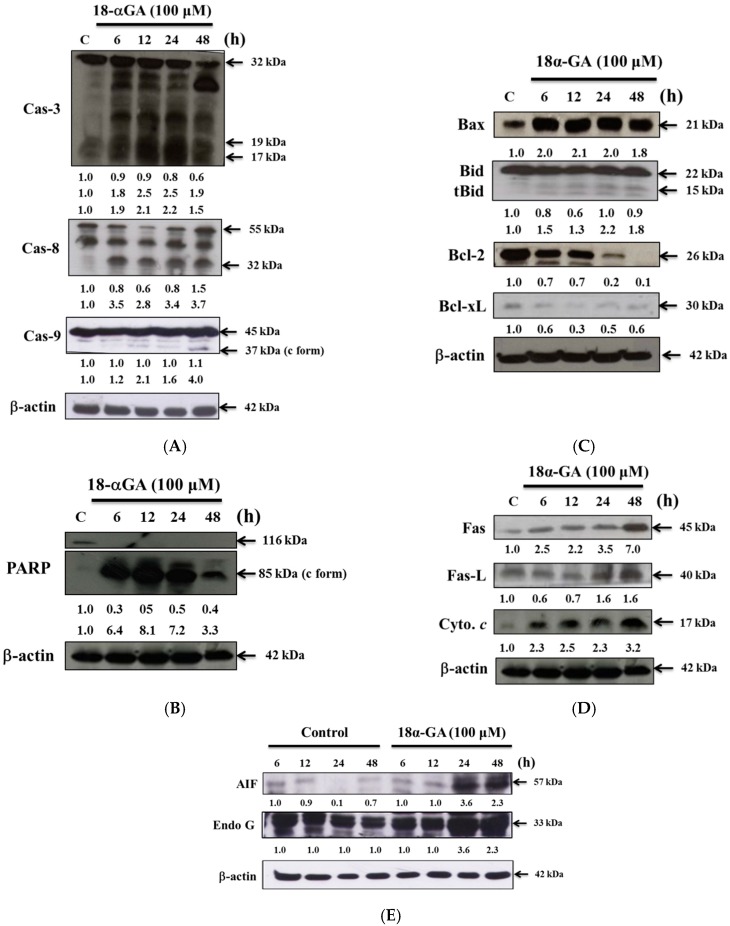
18α-GA affects apoptosis associated protein expression in HL-60 cells. Cells were treated with 18α-GA (100 μM) for 6, 12, 24 and 48 h and then apoptosis associated proteins were examined by western blotting as described in the Materials and Methods section. (**A**) active-caspase-8,-9 and -3; (**B**) PARP; (**C**) Bax, Bid, Bcl-2 and Bcl-xL; (**D**) Fas, Fas-L and cytochrome c (Figure 4D); (**E**) AIF and Endo G.

In order to examine 18α-GA-induced cell apoptosis via the alteration of apoptosis associated protein expression in HL-60 cells, cells were treated with 18α-GA (100 μM) for 6, 12, 24 and 48 h and then apoptosis-associated proteins were examined by western blotting (Figure 4). The results showed that 18α-GA significantly increased the expression of active-caspase-3, -8 and -9 (Figure 4A), cleaved-form-PARP (Figure 4B), Bax and t-Bid (Figure 4C), Fas, Fas-L and cytochrome c (Figure 4D), AIF and Endo G (Figure 4E) but decreased the anti-apoptotic proteins such as Bcl-2 and Bcl-xL (Figure 4C) in HL-60 cells that are associated with cell apoptosis. Those results indicate that 18α-GA induces apoptosis of HL-60 cells through surface death receptor and mitochondria-dependent pathways.

### 2.5. 18α-GA Altered the Translocation of Apoptotic Associated Proteins in HL-60 Cells

**Figure 5:**
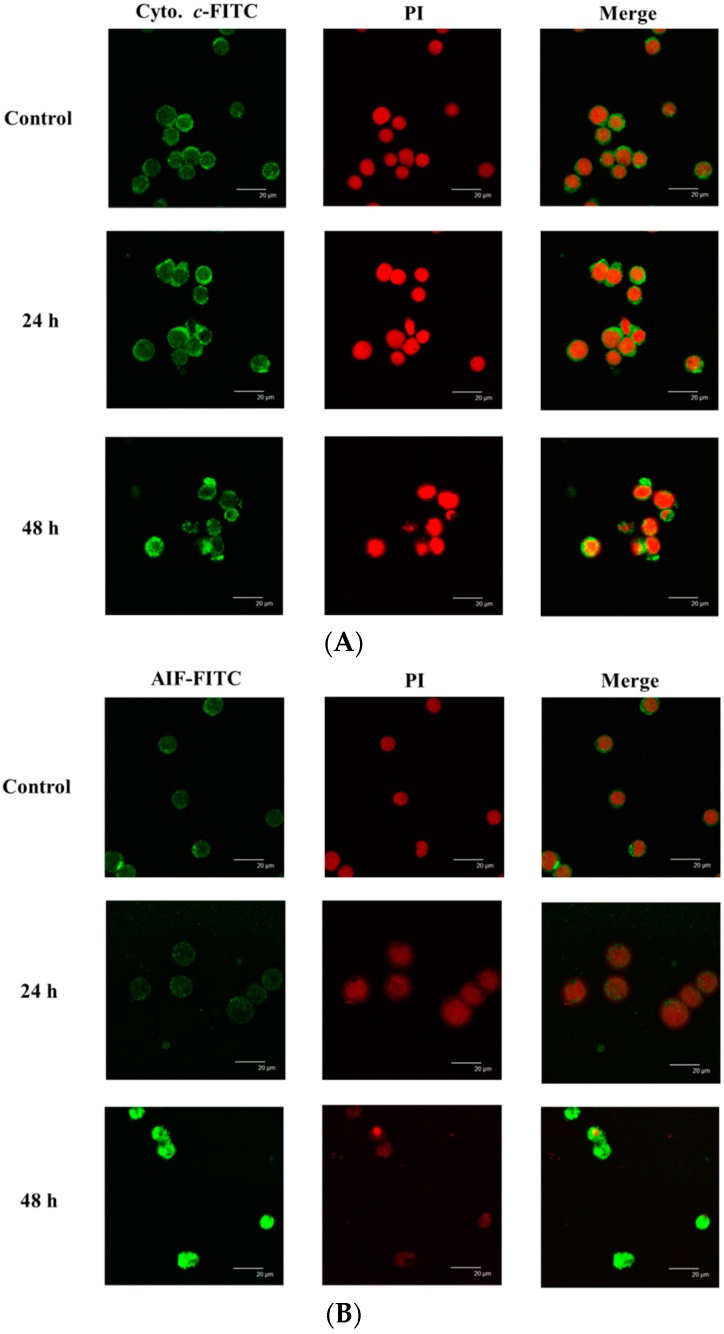
18α-GA affects the translocation of apoptotic associated proteins in HL-60 cells. Cells were treated with 100 μM of 18α-GA for 48 h and stained by anti- cytochrome c (**A**); and AIF (**B**) and then stained with secondary antibody and examined and photographed with a Leica TCS SP2 confocal laser microscopy system as described in the Materials and Methods section.

To further investigate how 18α-GA alters the translocation of apoptosis-associated proteins in HL-60 cells, we conducted confocal laser microscopy experiments (Figure 5). The results indicated that 18α-GA increased cytochrome c (Figure 5A) and AIF (Figure 5B) expression in HL-60 cells. These observations indicate that cytochrome c and AIF were released from the mitochondria into the cytoplasm compared to the control group.

## 3. Discussion

Numerous reports have shown that some clinical anticancer drugs could be obtained from natural products, thus, finding novel anticancer agents from natural plants has been recognized as one of the best strategies for the development of chemotherapeutic agents. Glycyrrhetinic acid (GE) can occupy mineralocorticoid and glucocorticoid receptors [29] with a measurable affinity for mineralocorticoid receptors in mononuclear leukocytes [30]. Furthermore, GE can block 11-β-hydroxysteroid dehydrogenase type 1, thus reducing the availability of cortisol at the adipocyte level [31]. Although several reports have demonstrated that 18α-GA induced cytotoxic effects on human cancer cells, there is no available information to show if 18α-GA induces cytotoxic effects on human leukemia HL-60 cells. In the present study, we examined the in vitro cytotoxicity of 18α-GA on HL-60 cells. Firstly, the anti-proliferation effects of 18α-GA on HL-60 cells were investigated and MTT assay results indicated that 18α-GA inhibited cell proliferation with an IC_50_ of 100 μM (Figure 1). Next, we examined 18α-GA-induced nuclear condensation, DNA damage and fragmentation and we found that 18α-GA induced nuclear condensation (Figure 2A), DNA damage (Figure 2B) and fragmentation (Figure 2C) that are the hallmarks of cell apoptosis, which means that 18α-GA induces cell apoptosis in HL-60 cells in vitro. Thirdly, we investigated 18α-GA induced cell apoptosis and whether or not it occurred through mitochondrial dysfunction and involved caspase activities and the results indicated that 18α-GA decreased the levels of ΔΨm (Figure 3A), and increased the activities of caspas-8, -9 and -3 (Figure 3B–D). Fourth, we investigated possible protein expression associated with cell apoptosis, thus, western blotting was used to examine the protein expression and the results indicated that 18α-GA increased caspase-8, -9 and -3 (Figure 4A) and cleaved-form-PARP (Figure 4B), increased pro-apoptotic proteins such as Bax, t-Bid and decreased anti-apoptotic proteins like Bcl-2 and Bcl-xL (Figure 4C) and induced Fas, Fas-L and cytochrome c (Figure 4D), and increased AIF and Endo G (Figure 4E). Fifth, we further investigated whether 18α-GA induced cytochrome c and AIF from mitochondria, which was assayed by confocal laser microscopy and results indicated that 18α-GA increased the releases of cytochrome c and AIF from mitochondria into the cytoplasm (Figure 5A,B).

Herein, we found that 18α-GA inhibited cell proliferation and led to a decrease in the total viability when compared to untreated groups, which is in agreement with other reports which demonstrated that 18α-GA inhibits the proliferation of activated hepatic stellate cells (HSCs) [26]. In order to investigate how 18α-GA reduced total viability and whether this went through the induction of apoptosis in HL-60 cells, we used a DNA gel electrophoresis assay which indicated that 18α-GA induced cell apoptosis based on the development of DNA fragmentation (DNA ladder) that is a characteristic of cell apoptosis and this finding also is in agreement with other reports which indicated that 18α-GA induced cell apoptosis in hepatic stellate cells (HSCs) [26].

It is well documented that anticancer drugs induced cell death through the induction of cell apoptosis which can go through mitochondrial dysfunction or decreased levels of ΔΨm [32,33]. Furthermore, Ca^2+^ accumulation can impair mitochondrial function and lead to increased release of reactive oxygen species (ROS) [34]. We used a flow cytometry assay and found that 18α-GA decreased the levels of ΔΨm in HL-60 cells (Figure 3A) and these effects are time-dependent. This pathway is named the intrinsic signaling pathway. It was reported that the ratio of Bax/Bcl-2 is involved in the levels of ΔΨm, so if this agent caused decreased levels of ΔΨm in mitochondria which can cause cytochrome c and/or AIF release, it could lead to cell apoptosis [35,36]. Thus, we examined associated protein expression and we found that 18α-GA increased the pro-apoptotic proteins such as Bax and t-Bid (Figure 4B) and decreased the anti-apoptotic proteins Bcl-2 and Bcl-xl (Figure 4C) and also increased cytochrome c and AIF (Figure 4E) in HL-60 cells. This finding is in agreement with reports which indicated that 18α-GA decreased the anti-apoptotic proteins such as Bcl-2 and Bcl-xL in human gingival fibroblasts [28]. Based on those findings, we suggest that 18α-GA induces apoptosis of HL-60 cells through a mitochondria-dependent pathway. We also used confocal laser microscopy to examine the release of apoptotic proteins from the mitochondria and the results indicated that 18α-GA increased cytochrome c (Figure 5A) and AIF (Figure 5B) release from mitochondria to the cytoplasm.

It also is well known that some anticancer drugs inducing cancer cell apoptosis are involved in the activation of caspases such as caspase-8, -9 and -3, therefore, some apoptotic pathways are also named caspases-dependent pathways [37]. We used flow cytometry to assay the activities in HL-60 cells after exposure to 100 μM of 18α-GA and we found that 18α-GA increased the activities of caspase-8, -9 and -3 (Figure 3B–D), which was also confirmed by western blotting (Figure 4A) which indicated that 18α-GA increased the active forms of caspase-8, -9 and -3 in HL-60 cells. It is well documented that cell apoptosis may go through the extrinsic death receptor pathway and/or the intrinsic mitochondrial pathway [38,39]. In the extrinsic pathway, Fas and Fas-L activate pro-caspase-8 and then activate downstream executioner caspases, including caspase-3, causing apoptosis or they also cleave Bid into truncated Bid this leads to mitochondrial dysfunction leading to cytochrome c release and induced caspase-9 and -3 activation to induce apoptosis, which is called the intrinsic pathway [12]. Those pathways also can be called caspases-dependent pathways. Furthermore, it is also well documented that agent-induced cell apoptosis may go through the decrease of the levels of ΔΨm and subsequent release of AIF and Endo G from the mitochondria into nuclei to induce apoptosis. It was reported that mTOR inhibitors have been used to treat several advanced cancers [40], thus, further investigations are needed regarding whether or not 18α-GA induced cancer cell apoptosis involves AKT/mTOR pathways.

**Figure 6:**
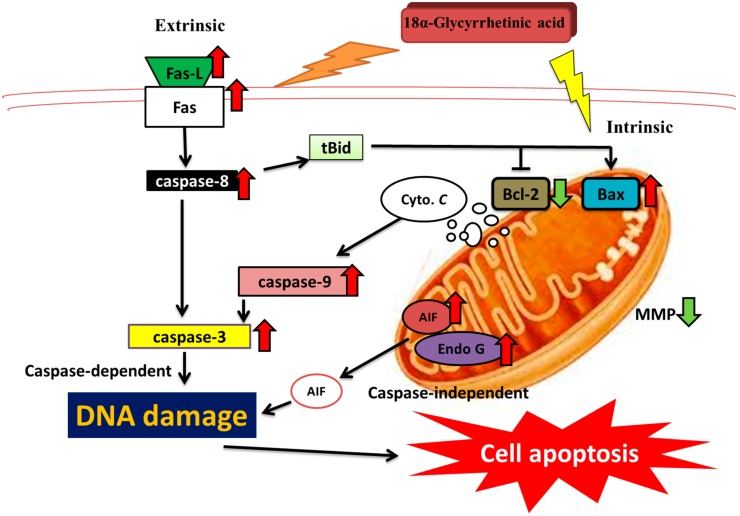
The possible signaling pathways for 18α-GA induced apoptosis in human leukemia HL-60 cells in vitro.

In summary, the present study demonstrated that 18α-GA inhibits the in vitro cell growth of HL-60 cells by inducing cellular apoptosis. Those findings also revealed that 18α-GA induced cell apoptosis was caspase-dependent and the mitochondria-dependent pathways were accompanied by the releases of cytochrome c, caspase-9 and -3 activation and/or AIF release. Overall the possible pathways for 18α-GA induced cell apoptosis are summarized in Figure 6. These findings indicate the therapeutic potential of 18α-GA in treating human leukemia.

## 4. Materials and Methods

### 4.1. Chemicals and Reagents

18α-Glycyrrhetinic acid (18α-GA), 4,6-diamidino-2-phenylindole (DAPI), dimethyl sulfoxide (DMSO), *N*-acetyl-l-cysteine (NAC), propidium iodide (PI) and trypsin-EDTA were obtained from Sigma Chemical Co. (St. Louis, MO, USA). RPMI-1640 medium, fetal bovine serum (FBS), l-glutamine and antibiotics (penicillin-streptomycin) were purchased from GIBCO^®^/Invitrogen Life Technologies (Carlsbad, CA, USA). Primary antibody against PARP, caspase-3,-8, -9, AIF, Endo G, Bid, Bax, Bcl-2, Bcl-xL, cytochrome c, Fas, Fas-L and peroxidase conjugated secondary antibodies were purchased from Cell Signaling (St Louis, MO, USA). 18α-GA was dissolved in DMSO. Cell culture grade DMSO was used for vehicle at 0.1%.

### 4.2. Cell Culture

The human leukemia HL-60 cell line was purchased from the Food Industry Research and Development Institute (Hsinchu, Taiwan). HL-60 cells were routinely maintained in RPMI-1640 medium supplemented with 10% FBS and antibiotics (100 units/mL penicillin, 100 μg/mL streptomycin and 2 mM glutamine) in a humidified atmosphere containing 5% CO_2_ at 37 °C [41,42].

### 4.3. Cell Proliferation Examination

Cytotoxicity of 18α-GA was evaluated by the MTT assay. HL-60 cells (1 × 10^4^ cells/well) were placed onto 96-well plates in 100 μM of RPMI-1640 medium. The medium contained 18α-GA (0, 40, 80, 120 and 160 μM) for 48 h. MTT (5 mg/mL, 20 μL) was added to each well for 4 h and then 100 μL/well of DMSO was added to dissolve the formazan. Optical density of each well was measured by a microplate reader (Bio-Rad 680, BioRad Inc., Hercules, CA, USA) at 570 nm as previously described [41,43,44].

### 4.4. Nuclear Staining with DAPI

Cells were placed in 6-well plate at a density of 2 × 10^5^ cells/well and cells were incubated with 18α-GA at concentrations ranging from 40–120 μM for 48 h. Cells were fixed in 3% formaldehyde for 20 min, and then washed with PBS. After disrupting with 0.1% Triton X-100/PBS for 20 min and then washed with PBS. Finally, we add the DAPI solution (1 μg/mL) at 37 °C without light. Nuclear morphologies were photographed using a fluorescence microscope (Eclipse TE 300, Nikon, Tokyo, Japan) as described previously [45].

### 4.5. Comet Assay

Cells were placed in 6-well plate at a density of 2 × 10^5^ cells/well and cells were incubated with 18α-GA ranging from 40–120 μM for 48 h. After treatment, cells from each treatment were harvested to examine the DNA damage with the Comet assay as described previously [45].

### 4.6. DNA Gel Electrophoresis

Cells (2 × 10^5^ cells/well) were maintained in 6-well plates and incubated with 18α-GA concentrations ranging from 40–120 μM for 48 h. Cells were harvested and DNA extracted by using DNA isolation kit as described previously [46]. DNA from each treatment (2 μg) was separated in 0.5% agarose gel as described previously [46]. Ethidium bromide was added to stain DNA and the intensity of the DNA bands was examined and photographed under a fluorescence microscope.

### 4.7. Measurement of the Levels of Mitochondrial Membrane Potential (ΔΨm)

Cells (2 × 10^5^ cells/well/6-well plate) were treated with 100 μM of 18α-GA for 0, 6, 12, 24 and 48 h. Cells from each treatment were harvested and washed twice with PBS, re-suspended in 500 μL of DiOC_6_ (4 mol/L) and were incubated at 37 °C for 30 min without light. After incubations, cells were measured for the levels of ΔΨ_m_ by flow cytometry (FACS Calibur, Becton Dickinson, San Joes, CA, USA) as described previously [41].

### 4.8. Measurements of Caspase-3, Caspase-8 and Caspase-9 Activities

Cells (2 × 10^5^ cells/well/6-well plate) were treated with 100 μM of 18α-GA for 0, 6, 12, 24 and 48 h. Cells were collected, re-suspended in 25 μL of 10 μM substrate solution containing CaspaLux8-L_1_D_2_ for caspase-8 activity measurement, or containing PhiPhiLux-G1D1 for caspase-3 activity measurement, or containing CaspaLux9-M1D2 for caspase-9 activity measurement before being incubated at 37 °C for 60 min. After incubations, cells were washed and caspase-8, -3 and -9 activities were analyzed by flow cytometry as described previously [41], or cells were pretreated with caspase-3 inhibitor (Z-EDVD-FMK) for 1 h [41] and then were treated with 100 μM of 18α-GA for 48 h and cell viability and caspase-3 activity was measured by flow cytometry.

### 4.9. Western Blotting Analysis

Cells (1 × 10^6^ cells/dish) were placed in 10 cm dish and treated with 100 μM of 18α-GA for 0, 6, 12, 24 and 48 h. Both adherent and floating cells were collected and lysed by lysis buffer and total protein was measured by Bio-Rad protein assay kit (Bio-Rad) as described previously [45]. The protein of total cells was separated by 12% (*v*/*v*) sodium dodecyl sulfate polyacrylamide gel electrophoresis (SDS-PAGE) and transferred onto polyvinylidene difluoride (PVDF) membranes (Millipore, Temecula, CA, USA) which were subsequently stained with the primary antibodies against caspase-8, -3 and -9, PARP, Bax, Bid, Bcl-2, Bcl-xL, Fas, Fas-L, cytochrome c, AIF, Endo G, and β-actin followed by a peroxidase conjugated secondary antibodies. Protein bands on the membrane were visualized and detected using an enhanced chemiluminescence using ECL detection system (Millipore) [45,46]. Protein expression was quantified by densitometry using Image J.

### 4.10. Confocal Laser Scanning Microscopy Assay

Cells (2 × 10^5^ cells/well) were placed in 6-well plate and were treated with 100 μM of 18α-GA for 48 h and then were fixed with 4% formaldehyde in PBS for 15 min. After fixation, cells were treated with 0.1% Triton-X 100 in PBS for 20 min and using 2% BSA for blocking non-specific binding sites, were washed with PBS twice and stained with primary antibodies anti-cytochrome c and -AIF (all in green fluorescence) for overnight. Cells were washed and were stained with secondary antibody (FITC-conjugated goat anti-mouse IgG) and PI (red fluorescence) for nuclein examinations. Slides were mounted, examined and photo-micrographed under a Leica TCS SP2 Confocal Spectral Microscope (Leica Microsystems, Heidelberg, Germany) as described previously [45].

### 4.11. Statistical Analysis

Values were presented as mean ± standard deviation (SD) from triplicate experiments. Statistical analysis of all data was conducted by Student’s *t* test. The *p* < 0.05 was considered statistically significant.
